# Profiling of adrenal corticosteroids in blood and local tissues of mice during chronic stress

**DOI:** 10.1038/s41598-023-34395-2

**Published:** 2023-05-04

**Authors:** Karla Vagnerová, Michal Jágr, Chahrazed Mekadim, Peter Ergang, Hana Sechovcová, Martin Vodička, Kateřina Olša Fliegerová, Václav Dvořáček, Jakub Mrázek, Jiří Pácha

**Affiliations:** 1 Institute of Physiology, Czech Academy of Sciences, Vídeňská 1083, 142 00 Prague 4—Krč, Czech Republic; 2 Quality and Plant Products, Crop Research Institute, Prague, Czech Republic; 3 Institute of Animal Physiology and Genetics, Czech Academy of Sciences, Prague, Czech Republic; 4 Faculty of Agrobiology, Food and Natural Resources, Department of Microbiology, Nutrition and Dietetics, Czech University of Life Sciences, Prague, Czech Republic; 5 Department of Physiology, Faculty of Science, Charles University, Prague, Czech Republic

**Keywords:** Stress and resilience, Neuroendocrinology, Adrenal cortex hormones, Microbiome

## Abstract

Stress increases plasma concentrations of corticosteroids, however, their tissue levels are unclear. Using a repeated social defeat paradigm, we examined the impact of chronic stress on tissue levels of corticosterone (CORT), progesterone (PROG), 11-deoxycorticosterone (11DOC) and 11-dehydrocorticosterone (11DHC) and on gut microbiota, which may reshape the stress response. Male BALB/c mice, liquid chromatography-tandem mass spectrometry and 16S RNA gene sequencing were used to screen steroid levels and fecal microbiome, respectively. Stress induced greater increase of CORT in the brain, liver, and kidney than in the colon and lymphoid organs, whereas 11DHC was the highest in the colon, liver and kidney and much lower in the brain and lymphoid organs. The CORT/11DHC ratio in plasma was similar to the brain but much lower in other organs. Stress also altered tissue levels of PROG and 11DOC and the PROG/11DOC ratio was much higher in lymphoid organs that in plasma and other organs. Stress impacted the β- but not the α-diversity of the gut microbiota and LEfSe analysis revealed several biomarkers associated with stress treatment. Our data indicate that social defeat stress modulates gut microbiota diversity and induces tissue-dependent changes in local levels of corticosteroids, which often do not reflect their systemic levels.

## Introduction

The response to stressful physical and psychological challenges represents adaptive processes to protect the survival of individuals and maintain their body systems under the current conditions^1^. Stressful stimuli induce a cascade of events in the hypothalamic–pituitary–adrenal (HPA) axis, which leads to the activation of the adrenal cortex and the release of glucocorticoids, cortisol in humans and corticosterone (CORT) in rats and mice. Although the neural pathways of stress responses in the brain differ depending on the type of stressor, activation of the HPA axis is thought to be a final common pathway, which results in a net increase in circulating glucocorticoids^2^. Glucocorticoids act on many tissues to induce genomic and nongenomic effects that regulate metabolic, immune, cognitive and reproductive systems^3^. In addition to glucocorticoids, stress also results in a rapid increase of progesterone (PROG) and 11-deoxycorticosterone (11DOC) secretion from the adrenal cortex^4,5^.

The local effect of glucocorticoids does not depend only on their plasma concentrations because some tissues can actively regulate local steroid levels. Enzymes of glucocorticoid de novo synthesis are expressed not only in the adrenal cortex but also in other tissues such as intestine^6–8^, lymphoid organs^9–11^ and immune cells^12,13^, although only the first steps of steroid synthesis have been found in the latter. Additionally, some cells express the enzyme 11-hydroxysteroid dehydrogenase type 1 (11HSD1), which converts inactive 11-oxo derivatives of glucocorticoids, cortisone and 11-dehydrocorticosterone (11DHC), into active glucocorticoids cortisol and CORT and thus effectively amplifies the local level of active glucocorticoids. This enzyme is expressed in many tissues, including the liver, kidney, intestine^14^, lymphoid organs^15,16^ and immune cells^17^. Within some tissues such as the kidney and intestine, CORT and cortisol can also be locally converted to their inactive 11-oxo derivatives, 11DHC and cortisone, by the enzyme 11-hydroxysteroid dehydrogenase type 2 (11HSD2)^14^. In addition to peripheral tissues, the brain also shows local production of steroids via de novo synthesis or via local conversion of steroids originating in peripheral endocrine organs, including the adrenal gland^18,19^. Interestingly, stress not only increases the systemic level of glucocorticoids but also regulates steroidogenic enzymes, including 11HSD1, in a time- and tissue-dependent manner^20–22^ suggesting that local production and metabolism of corticosteroids and their changes in response to stress may modulate the effects of the HPA axis response in particular tissues.

Although the enzymes of extraadrenal steroidogenesis and 11HSDs are assumed to shape the local corticosteroids, profiling of tissue corticosteroids have never been directly quantified in response to chronic stress except for CORT concentration in the brain, thymus and intestine of chronically stressed rats and mice^23,24^. Therefore, the present study aimed to compare the systemic and local levels of pregnenolone (PREG), PROG, 11DOC, CORT and 11DHC in the blood, brain and peripheral tissues of mice exposed to chronic stress using the model of repeated social defeat. Because a number of studies have indicated that chronic exposure to stress changes the structure of the gut microbial community^25^ and that the gut microbiota can alter the behavior and the stress responses of the host^26^ and modulate the tissue levels of some steroids^27^, we also determined whether exposure to chronic psychosocial stress changed the community structure of the microbiota in experimental animals.

## Materials and methods

### Animals and repeated social defeat stress

The experiments were performed on 9-week-old male BALB/c mice (Institute of Physiology, Czech Academy of Sciences, Prague), which were housed in a temperature-controlled room (22 ± 1 °C) on a 12/12-h light/dark cycle (lights on at 7 a.m.) with free access to pellet diet Altromin 1314 (Altromin, Lage, Germany) and tap water. After two weeks of acclimation period the mice were randomly divided into two groups (stressed and control group; n = 5 animals per group) and housed individually in the cages. The social defeat stress protocol used in this study was similar to the resident-intruder paradigm used in our previous work^22^. Briefly, the protocol was based on the fact that a male mouse defends its territory against an unfamiliar male intruder and that exposure of a younger intruder to the resident represents an unpredictable allostatic load associated with activation of the HPA axis. Older sexually experienced males (n = 9) were used as residents that were housed individually for 7 days before the experiment without a change of bedding. Following the seven-day isolation period, each intruder was exposed three times per week (every second day) to a different resident. Each intruder was exposed overall 16 times to the resident. First, the resident and intruder were allowed to stay in direct physical contact with each other in the resident’s cage for 10 min, after which the intruders were separated by a steel mesh to preserve sensory contact between resident and intruder for the next 50 min. This setup reduced risk of injuries, while the intruder was subjected to continuous psychological stress due to sensory interaction with the resident. After the social interaction session, the intruder was removed from the resident's cage and returned to its home cage located in the same room. Control (unstressed) mice remained undisturbed in their home cages in a separate quiet room and sacrificed one day before stress group. To minimize the effect of circadian rhythm, all stress sessions were carried out between 9 and 10:30 a.m. except the last one that started at 8:55 a.m. with 15 min gaps between respective mice. Every mouse was sacrificed exactly 80 min after the stressor initiation (20 min after last stress session). Both groups of mice (stressed as well as control group) were sacrificed between 10:15 and 11:40 a.m. The experiments were approved by the Animal Care and Use Committee of the Institute of Physiology v.v.i., Czech Academy of Sciences.

### Tissue sampling and processing

After the last social interaction session, mice were immediately anesthetized with isoflurane vapor. This type of anesthesia was chosen because isoflurane anesthesia did not have significant impact on CORT levels during sacrifice^28^. Control mice were carefully removed from their home cage and deeply anesthetized within 1 min from disturbance of their home cage. Blood was collected by cardiac puncture, centrifuged at 2000 *g* for 10 min at 4 °C, and stored at − 80 °C. Anesthetized mice were then decapitated and brain (cerebral cortex, hippocampus), liver, kidney, colon, thymus, and mesenteric lymph node (MLN) samples were harvested, snap frozen in liquid nitrogen and stored at − 80 °C until further analysis. Samples of fecal pellets were collected before the start of the stress period and immediately after its end, placed into sterile tubes and stored at − 80 °C until the extraction of genetic material for sequencing.

Steroids were extracted twice from blood and tissue homogenates prepared in 1 ml of ice-cold water with a Polytron homogenizer (Kinematica AG, Luzern, Switzerland). Blood (100 μl) plus 900 μl of water or homogenate samples (1000 μl) were extracted by adding 2 ml of tert-butyl methyl ether. The mixture was vortexed (1500 rpm) 3 times for 30 s and centrifuged at 1500 *g* for 15 min to separate the water and organic phases. The centrifuged samples were left to freeze, and the upper organic phase was transferred to a clean tube. The water phase was re-extracted again with 2 ml of tert-butyl methyl ether, and the organic phases were combined, evaporated in a nitrogen stream at 40 °C and stored at − 80 °C before further processing for LC–MS/MS analysis. The protein concentration of tissue homogenates was measured in 10 μl of tissue homogenate using the bicinchoninic acid method.

### Liquid chromatography: tandem mass spectrometry (LC–MS/MS)

Reference standards of each steroid compound (PREG PROG, 11DOC, CORT and 11DHC) and internal standards (PROG-*d*_*9*_, CORT-*d*_*4*_) were dissolved in methanol to obtain stock solutions of 0.5 mg/ml and stored up to a week at − 18 °C. Internal standards were then added to the individual or quality control test samples to a final concentration of 0.1 µg/ml. Calibration curves for the steroid compound quantification were constructed by plotting the peak area (adjusted by an internal standard) vs. the concentration of relevant reference standards. Curves were made by using six calibration points in the concentration range of 0.0001–1.000 µg/ml (including control sample). In this range, the response of mass detector vs. steroid concentration was linear. Prior to LC–MS/MS analysis, dried supernatants from each sample were redissolved in 150 µl of methanol containing internal standards using an ultrasonic bath, centrifuged (15 min; 13,500 rpm, room temperature) and filtered through 0.2 µm PVDF spin filters (Thermo Fisher Scientific, Rockwood, TN, USA). The extracts were transferred to vials and stored at − 18 °C prior to LC–MS/MS analysis.

Steroid analysis by LC–MS/MS was conducted as previously described^7^. Briefly, chromatographic separation was carried out on an ultrahigh-performance liquid chromatographic system Dionex UltiMate 3000 (Dionex Softron GmbH, Germering, Germany) using a reversed-phase Kinetex XB-C18 column (2.1 × 100 mm, 2.6 µm; Phenomenex, Torrance, CA, USA) and gradient elution. The mass spectrometric analysis was processed using a quadrupole/orbital ion trap Q Exactive mass spectrometer (Thermo Fisher Scientific, San Jose, CA, USA) equipped with a heated electrospray ionization source (HESI-II) and Xcalibur software, version 4.0. The mass spectrometer was operated in positive ion mode with the source conditions: spray voltage 2.5 kV; sheath gas flow 49 AU; auxiliary gas flow 12 AU and sweep gas flow 2 AU; capillary temperature 260 °C; heater temperature 419 °C. Data were acquired using Xcalibur software. Quality control samples were entered after every tenth analysis to ensure proper performance of LC–MS/MS analyses. The measurements of samples were done in three replicates. Concentrations of steroids in the samples were extrapolated from calibration curves and converted to ng per ml (for plasma) or normalized to mg of protein of the tissue. A steroid concentration was considered undeterminable if it was below the lowest value on the calibration curve (BC).

The relative recovery of the steroids from the tissues was estimated by comparing the peak area of analytes in the tissue samples spiked with standard solutions of CORT, 11DHC, 11DOC, PROG and pregnenolone (PREG) at concentrations of 300, 30, 3 and 0.3 ng/ml before and after extraction. The mean relative recovery of steroids in the studied tissue samples ranged from 75 to more than 90% depending on the steroids and the matrices (PREG, 81.3 ± 5.0%; PROG, 86.2 ± 2.5%; 11DOC, 93.5 ± 3.2%; CORT, 93.2 ± 4.9%; 11DHC 77.6 ± 2.9%).

### Fecal microbiota sequencing analysis

To determine the microbiome profiles of the stressed and unstressed animals, fecal samples were collected aseptically in sterile tubes from individual mice immediately after voluntary defecation and stored at − 80 °C. Control samples were collected before the stress period (STAEX group; n = 10) and then 1 month later in unstressed mice (CTRL group; n = 5) and stressed animals after the last session on the day of sacrifice (STRESS group; n = 5). Total DNA was extracted from each stool sample using a QIAmp PowerFecal DNA Kit (Qiagen, Hilden, Germany) according to the manufacturer’s protocol. Extracted DNA was exploited for sequencing and microbiome analysis as mentioned previously^29^. Briefly, the amplicons of V4V5 region of the 16S rRNA gene were prepared from the extracted DNA. Libraries were prepared using NEBNext Fast DNA Library Prep Set (New England Biolabs, Ipswich, MA, USA), purified and quantified. Next Generation Sequencing was performed via the Ion Torrent platform (Thermo Fisher Scientific, Waltham, MA, USA) and the sequencing data are deposited in the GenBank (accession no. PRJNA896122). The sequencing was not successful for 3 samples from STAEX group, therefore the subsequent data analysis was performed only from 7 samples from this group (n = 7).

### Data analysis

The statistical analyses were carried out with Statistica software (StatSoft Inc. Tulsa, OK, USA), and the data are expressed as the mean ± SEM. Datasets were analyzed by one-way or two- way analysis of variance (ANOVA) followed by post hoc Tukey’s test. One-way ANOVA was used to compare the tissue profiles of steroid levels and CORT/11DHC, PROG/11DOC, CORT/PROG and CORT/11DOC ratios, whereas the effect of repeated social defeat stress on steroid levels in individual tissues was analyzed by unpaired Student’s t-test. Two-way ANOVA was used to reveal whether steroid levels in the adrenal gland and plasma differ by stress and steroid type and whether there is a stress x steroid type interaction. A P value ≤ 0.05 was regarded as significant.

Microbiome analysis was performed as previously described^24^. Briefly, bacterial 16S rDNA gene sequences obtained in FASTQ format were analyzed by the QIIME 2 version 2020.2 pipeline. The quality control was performed on demultiplexed sequences using DADA2 by filtering out chimeric sequences. Subsequently, clustering and taxonomy classification was assessed using SILVA database with 99% OTUs reference sequences. The α-diversity (describing the species diversity/richness in one ecosystem/group of control or stressed mice) was determined using Shannon diversity index based on the Kruskal–Wallis test. Principal Coordinate Analysis (PCoA) based on Bray–Curtis distance (β-diversity; describing the species diversity between two groups/control and stressed mice) was generated after rarefaction. The box plots for Shannon index of diversity and the 2-dimensional PCoA plots were generated in R-Studio (version 3.6.3) (http://www.rstudio.com/) using ggplot2 (https://ggplot2.tidyverse.org) packages. Ellipses mark 95% of confidence for each group and P value ≤ 0.05 was considered statistically significant. Adonis permutational multivariate analysis (Adonis/PERMANOVA) and Bray–Curtis distance matrix were used to evaluate the dissimilarity among samples with permutation set at 999. The linear discriminant analysis (LDA) with effect size (LEfSe) algorithm was performed in Galaxy module (http://huttenhower.sph.harvard.edu/galaxy) based on the factorial Kruskal–Wallis test and the pairwise Wilcoxon test to identify genera with significant differential relative abundances between stressed and control groups with α value 0.05 and threshold value 2.0 on the logarithmic LDA scores for discriminative features.

### Ethics declaration

The study was conducted according to the guidelines of the Declaration of Helsinki and approved by the Committee for the Protection and Use of Experimental Animals of the Institute of Physiology v.v.i., Czech Academy of Sciences. All experiments were performed in accordance with the relevant guidelines and regulations. The study is reported in accordance with the ARRIVE guidelines.

## Results

### Effect of repeated social defeat stress on adrenal and plasma levels of steroids

**Figure 1:**
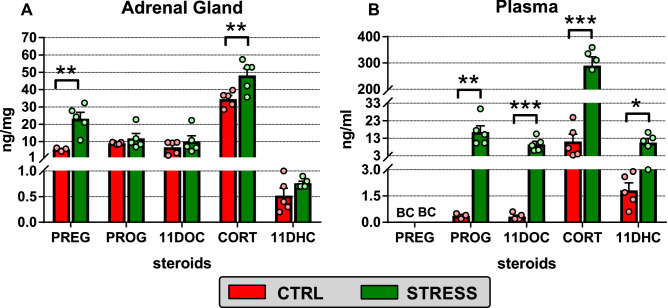
Effect of social defeat on the adrenal (**A**) and plasma levels (**B**) of pregnenolone (PREG), progesterone (PROG), 11-deoxycorticosterone (11DOC), corticosterone (CORT) and 11-dehydrocorticosterone (11DHC). Red columns, control unstressed mice (CTRL); green columns, mice exposed to chronic social defeat (STRESS); BC, bellow the lowest value of the calibration curve. The amounts of steroids were converted to ng per ml of plasma or ng per mg of protein for the adrenal gland. Data are shown as means ± SEM (n = 3–5). Significantly different values between stressed and unstressed mice: ^***^P < 0.001, ^**^P < 0.01, ^*^P < 0.05.

Steroid profiling in the adrenal glands of socially defeated and unstressed mice is summarized in Fig. 1A and Supplementary Fig. S1 online. A two-way ANOVA revealed a significant main effect of stress (F_1,48_ = 19.12, P < 0.001), steroid type (F_5,48_ = 78.62, P < 0.001) and stress x steroid type interaction (F_5,48_ = 3.99, P < 0.01). Tukey’s post hoc test indicated significant upregulation of adrenal PREG and CORT without any significant changes in the levels of PROG and 11DOC. Similarly, the local levels of 11DHC, which is generated from CORT by 11HSD2, did not differ between the adrenal glands of stressed and unstressed mice.

As expected, the plasma levels of corticosteroids increased significantly during social defeat regardless of steroid type except PREG (Fig. 1B; Supplementary Fig. S1 online). Two-way ANOVA showed a significant effect of stress (F_1,30_ = 76.03, P < 0.001), steroid type (F_3,30_ = 68.52, P < 0.001) and stress x steroid type interaction (F_3,30_ = 59.10, P < 0.01). In contrast to the adrenal glands, stress significantly upregulated not only plasma CORT (× 26) but also PROG (× 45), 11DOC (× 27) and 11DHC (× 6).

### Changes in corticosteroid levels in the brain and peripheral tissues of stressed mice

To identify changes in tissue levels of corticosteroids in peripheral tissues during stress, CORT, PROG, 11DOC and 11DHC were quantified in brain, colon, liver, kidney and lymphoid organs. In keeping with plasma, social defeat exposure increased local levels of corticosteroids, and this increase depended on the particular tissue.

**Figure 2:**
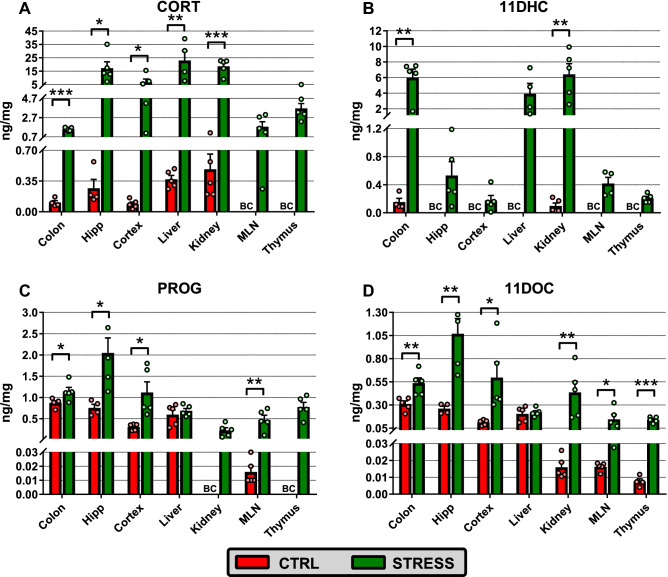
Effect of social defeat on profiling of (**A**) corticosterone (CORT), (**B**) 11-dehydrocorticosterone (11DHC), (**C**) progesterone (PROG) and (**D**) 11-deoxycorticosterone (11DOC) in the brain and peripheral tissues. Data are means ± SEM (n = 4–5). Red columns, control unstressed mice (CTRL); green columns, mice exposed to chronic social defeat (STRESS); BC, bellow the lowest value of the calibration curve; *Hipp* hippocampus, *MLN* mesenteric lymph node. The amounts of steroids were converted to ng per mg of protein. Significantly different values between stressed and unstressed mice: ***P < 0.001, **P < 0.01, *P < 0.05.

In control unstressed mice, local CORT was detected in all tissues with the exception of lymphoid organs. Stress significantly increased CORT levels in all tissues including the MLN and thymus (Fig. 2A; Supplementary Fig. S1 online). This increase was nearly 20 times in the colon, 40 times in the kidney and more than 60 times in the liver and brain. One-way ANOVA showed different levels of CORT across the tissues in both unstressed (F_4,18_ = 3.23, P < 0.05) and stressed (F_6,27_ = 7.73, P < 0.001) animals. In unstressed animals, post hoc comparison revealed that CORT levels in the kidney were significantly higher than the CORT levels in the colon (P < 0.05) and cortex (P < 0.01). For stressed animals, CORT levels were significantly higher in the hippocampus, liver and kidney than in other tissues (P < 0.05 or P < 0.01). In contrast to CORT, 11DHC in unstressed animals was detected only in the kidney and colon (Fig. 2B, Supplementary Fig. S1 online) without any significant differences between both tissues. Social defeat increased 11DHC levels in all studied tissues; however, there were significant differences between the tissues (one-way ANOVA, F_6,25_ = 11.41, P < 0.001); 11DHC levels were higher in the colon, kidney and liver than in other tissues (P < 0.001 or P < 0.05).

Similar to CORT, stress significantly increased the tissue levels of its precursors PROG and 11DOC (Fig. 2C,D; Supplementary Fig. S1 online). For unstressed mice, one-way ANOVA showed significantly different tissue levels of PROG (one-way ANOVA, F_4,19_ = 28.72, P < 0.001), with lower levels in the cortex and MLN than in the colon, hippocampus and liver (P < 0.05 or P < 0.001). The effect of stress was evident in all tissues except the liver and the differences between tissue levels of PROG persisted in stressed animals (one-way ANOVA, F_6,27_ = 12.73, P < 0.001). PROG levels in the hippocampus were significantly higher than the PROG levels in all other tissues (hipp. vs. colon or cortex: P < 0.05; hipp. vs. liver, kidney or MLN: P < 0.001; hipp. vs. thymus P < 0.01). Regarding 11DOC, stress significantly increased local levels of this steroid in all tissues, with the exception of the liver (Fig. 2D). Profiling of 11DOC tissue level was very similar to PROG (one-way ANOVA; unstressed mice: F_6,27_ = 33.26, P < 0.001, stressed mice: one-way ANOVA, F_6,28_ = 10.17, P < 0.001) and 11DOC level in hippocampus of stressed mice was significantly higher than in all other tissues (hipp. vs. colon or cortex: P < 0.05; hipp vs. liver, kidney, MLN or thymus P < 0.001).

**Figure 3:**
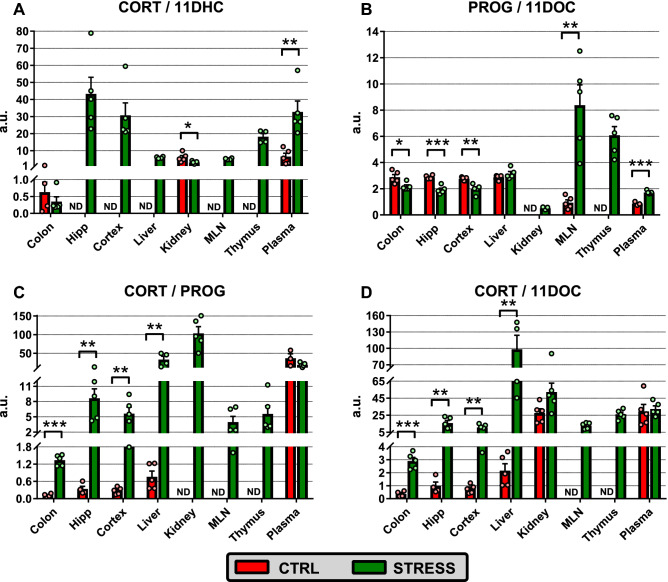
Effect of social defeat on profiling of (**A**) CORT/11DHC, (**B**) PROG/11DOC, (**C**) CORT/PROG and (**D**) CORT/11DOC ratio in the brain, peripheral tissues and plasma. Data are means ± SEM (n = 3–5). Red columns, control unstressed mice (CTRL); green columns, mice exposed to chronic social defeat (STRESS); ND, not determined; Hipp, hippocampus; MLN, mesenteric lymph node; CORT, corticosterone; 11DHC, 11-dehydrocorticosterone; PROG, progesterone; 11DOC, 11-deoxycorticosterone. Significantly different values between stressed and unstressed mice: ***P < 0.001, **P < 0.01, *P < 0.05.

To explore the functional relevance of 11HSD1 and 11HSD2 in brain and peripheral tissues, the ratio of CORT/11DHC was analyzed (Fig. 3A; Supplementary Fig. S2 online). In unstressed animals, this ratio was similar in plasma and kidney but was 10 times lower in colon (one-way ANOVA, F_2,11_ = 4.83, P < 0.05). Regarding stressed animals, the ratio CORT/11DHC differed significantly among the tissues (one-way ANOVA, F_7,29_ = 9.76, P < 0.001). The ratio was significantly lower in the colon, liver, kidney and MLN than in the plasma and brain (P < 0.05 or P < 0.01). Moreover, stress significantly elevated the CORT/11DHC ratio in plasma (P < 0.01) and decreased it in the kidney (P < 0.05).

It is well known that besides the adrenal gland there are tissues that express only some enzymes from the entire cascade of de novo synthesis of corticosteroids^18,19^. Therefore, we decided to calculate also other steroids ratios in individual tissues (Fig. 3; Supplementary Fig. S2 online). Stress significantly decreased the PROG/11DOC ratio in the colon, hippocampus and cortex but increased the ratio in MLN and plasma; stress did not change the ratio in the liver (Fig. 3B). Profiling of the PROG/11DOC ratio showed significant tissue differences both in unstressed (one-way ANOVA, F_6,22_ = 51.67, P < 0.001) and stressed animals (one-way ANOVA, F_7,31_ = 18.93, P < 0.001). In stressed animals, the PROG/11DOC ratio was significantly higher in lymphoid organs than in other tissues (P < 0.001), whereas in unstressed mice, this ratio was significantly lower in the MLN and plasma than in other tissues (P < 0.001).

Compared to CORT/11DHC and PROG/11DOC ratios, stress significantly increased both CORT/PROG and CORT/11DOC ratios in the colon, brain and liver (P < 0.01 or P < 0.001) with no effect in the plasma or in the plasma and kidney, respectively. (Fig. 3C,D; Supplementary Fig. S2 online). In unstressed mice, both ratios were more than 13 times higher in plasma (plasma and kidney for CORT/11DOC) than in the liver, brain and colon (CORT/PROG: one-way ANOVA, F_4,16_ = 15.42, P < 0.001; CORT/11DOC: one-way ANOVA, F_5,22_ = 11.64, P < 0.001). In contrast, the CORT/PROG ratio in the stressed mice reached the highest value in the kidney (P < 0.001), whereas the CORT/11DOC ratio was the highest in the liver (liver vs. kidney P < 0.05; liver vs. other tissues P < 0.001) (CORT/PROG: one-way ANOVA, F_7,31_ = 23.49, P < 0.001; CORT/DOC: one-way ANOVA, F_7,31_ = 12.14, P < 0.001).

### Effect of repeated social defeat stress on the gut microbiota

**Figure 4:**
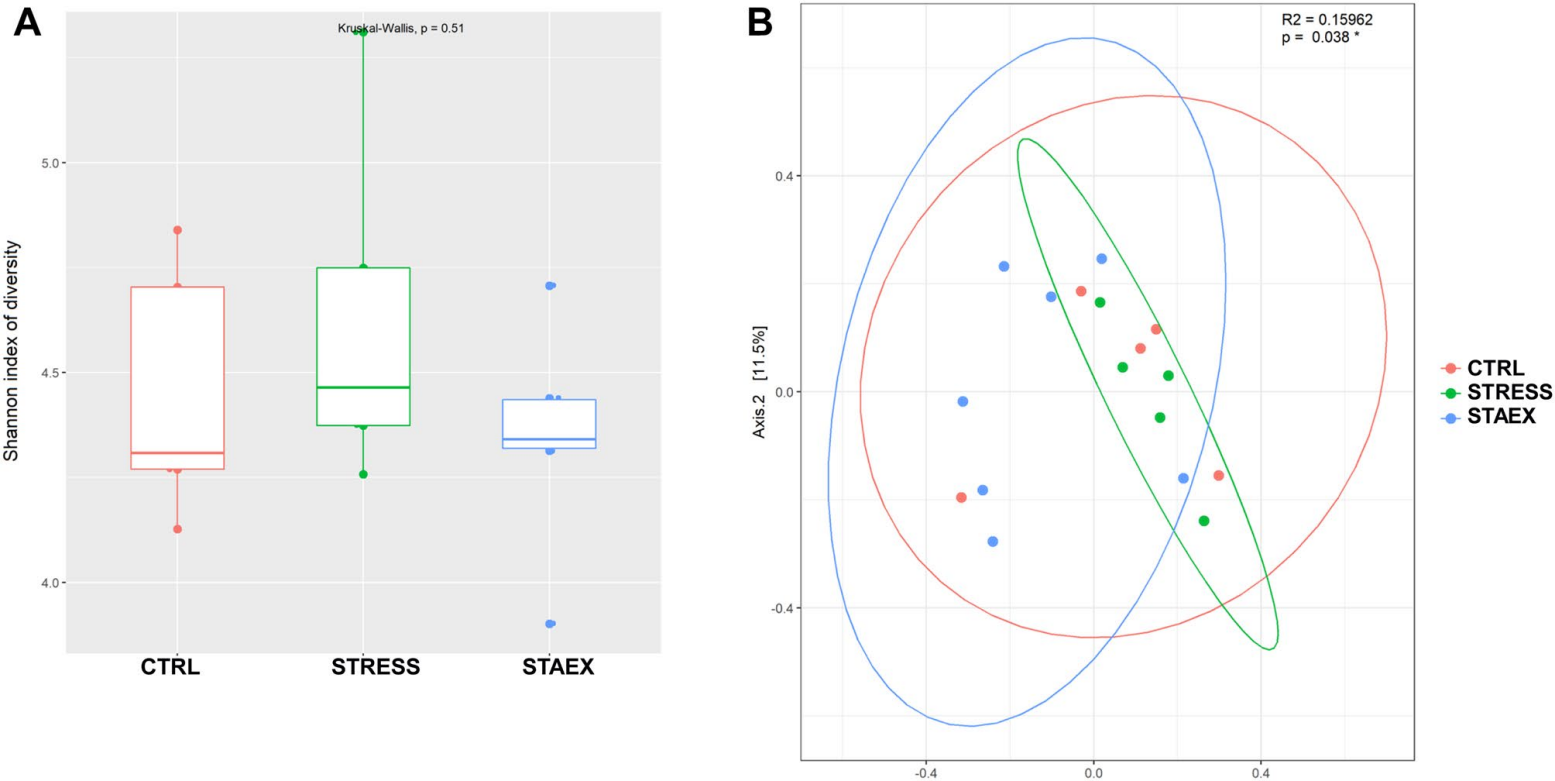
Effect of social defeat on the diversity of gut microbiota composition. (**A**) Box-plots illustrating α-diversity by Shannon index in bacterial microbiomes among different groups (Kruskal–Wallis test, P = 0.51). (**B**) Principal coordinate analysis plot (PCoA) based on the Bray–Curtis distance showed distinct clusters among different groups. The confidence level of the ellipse was 95%. STAEX, control samples collected before stress period (n = 7); CTRL, samples from non-stressed mice collected 1 month later then samples from STAEX group (n = 5); STRESS, samples from animals stressed for a period of 1 month.

**Figure 5:**
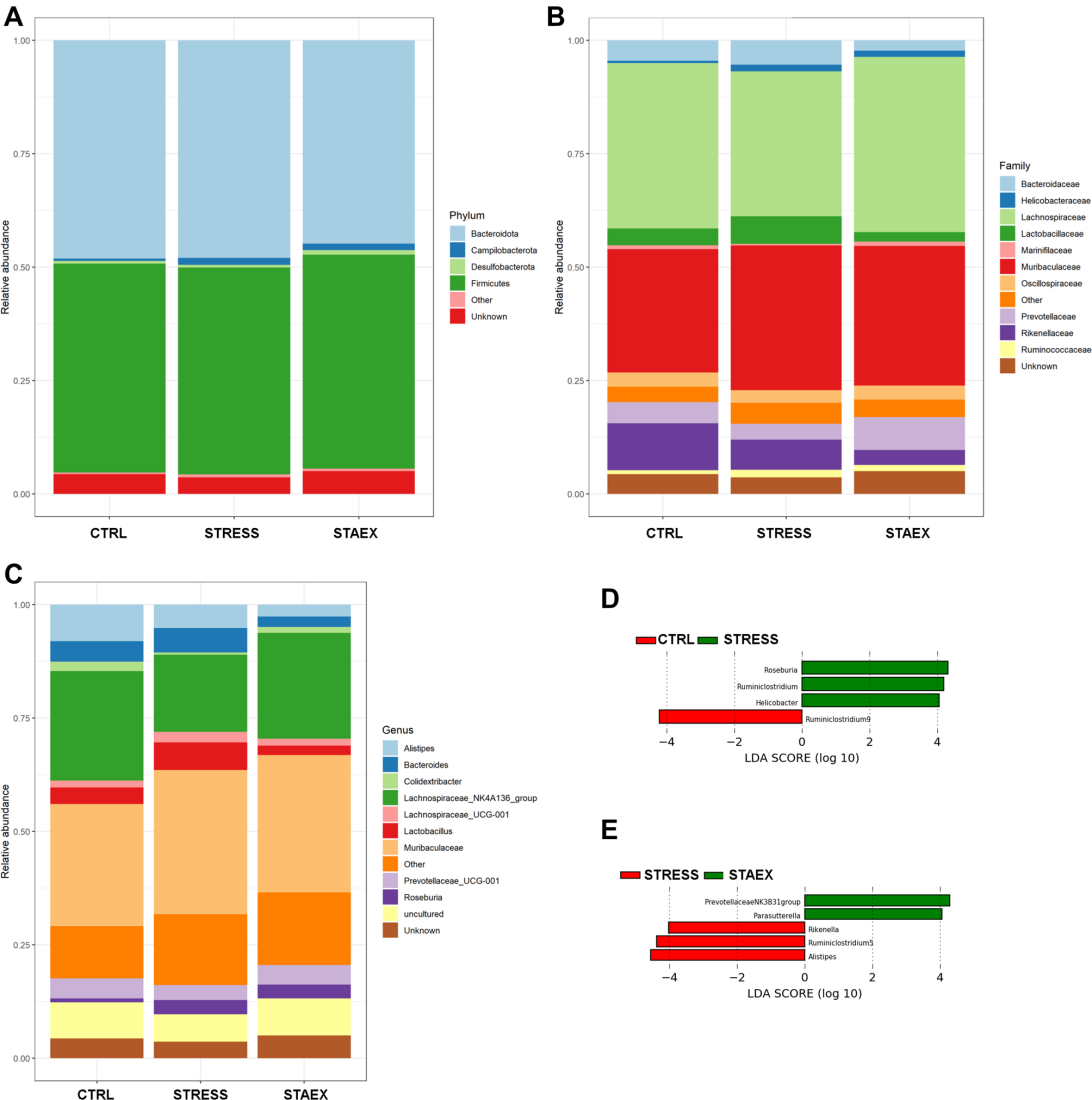
Effect of social defeat on the relative abundance of gut microbial populations at the levels of phylum (**A**), family (**B**), and genus (**C**). The linear discriminant analysis effect size (LEfSe) at genera levels in gut microbiome between stressed (STRESS group; n = 5) and control animals (CTRL group; n = 5) (**D**) and between stressed animals (STRESS group) and animals before the stress treatment (STAEX group; n = 7) (**E**).

For α-diversity, the Kruskal–Wallis test revealed no statistically significant differences in the Shannon index (P = 0.51) among the three groups (Fig. 4A). PCoA based on Bray Curtis distance (α-diversity) showed a significant stress effect on microbiome diversity (R^2^ = 0.160, P = 0.038). As shown in Fig. 4B, the cluster of stressed mice was centroid, whereas the clusters of the remaining two groups were largely dispersed. The relative composition of fecal microbiota at the phylum and family levels was similar in all mouse groups, and there was no marked difference between the control and stressed groups (*P* > 0.05). The major abundant phyla were *Bacillota* (previously known as *Firmicutes*) and *Bacteroidota* (previously known as *Bacteroidetes*), whereas *Lachnospiraceae* and *Muribaculaceae* were the most abundant families (Fig. 5A,B). At the genus level, *Muribaculaceae* (previously known as S24-7) and *Lachnospiraceae*_NK4A136_group were the highest abundant genera in all mice groups (Fig. 5C). To elucidate the specific taxa in the fecal microbiome in the stressed and unstressed groups, the LEfSe (LDA score > 2) method was used. According to this analysis (Fig. 5D,E), five bacterial genera were related to stress in the fecal microbiota: *Alistipes, Rikenella, Ruminiclostridium 5, Roseburia,* and *Helicobacter,* whereas *Ruminoclostridium 9* was the only genus related to unstressed mice (CTRL group). The genera from the *Prevotellaceae* NK3B31 group and *Parasutterella* were associated with the fecal microbiome of mice before stress treatment (STAEX group).

## Discussion

We have shown that: (*i*) the levels of CORT, 11DHC, PROG and DOC vary according to the tissues, (*ii*) the local tissue levels do not always change in parallel with the systemic plasma level, and (*iii*) the steroid profile differs between stressed and unstressed mice. In stressed animals, CORT levels were increased 19–75 times in local tissues compared to a 26-fold increase in plasma. PROG and 11DOC levels were 1–30 times higher upon stress in local tissues, whereas the plasma level of PROG was increased 45 times and 11DOC 27 times. Similar upregulation of CORT, PROG and 11DOC was observed recently in brain and thymus of prepubertal mice exposed to acute stress, although the stimulatory effect of stress was less pronounced compared to adult animals^30,31^. 11DHC, a well-known metabolite of 11HSD2, was not detected in most tissues of unstressed animals, with the exception of kidney, colon and plasma, in which stress increased 11DHC levels 65-, 40- and sixfold, respectively. The nondetectable levels of 11DHC in brain areas and lymphoid organs of unstressed mice are inconsistent with the data of Hamden et al.^18^ and Salehzadeh et al.^32^ who reported detectable levels of this steroid in brain and thymus. The reasons for this discrepancy are unknown but difference in the sensitivity and linearity of the methods used might be one of them. In addition, the measurable amounts of 11DHC were detected in the colon and kidney of unstressed controls, i.e. in the tissues, which express high 11HSD2 dehydrogenase activity^14^ and where higher local concentrations of 11DHC can be expected. Although 11HSD2 is expressed predominantly in mineralocorticoid target tissues such as the kidney and colon^14^, the expression of this enzyme at the mRNA or protein level was also demonstrated in some other organs, including the thymus, MLN and spleen, often without clear functional importance^32–34^. Thus, 11HSD2 might be inactive under basal conditions in the hippocampus, cortex and lymphoid organs, or 11DHC of blood origin might be rapidly metabolized by 11HSD1 in these tissues. High activity of 11HSD1, which regenerates CORT from 11DHC, was shown in the liver, but 11HSD1 was also found in the brain, thymus, spleen and MLN^14,16,33^.

Stress not only increased plasma and tissue levels of CORT above the values of unstressed controls, but the levels in the hippocampus, liver and kidney were also higher than in other tissues. These findings suggest tissue-specific differences of CORT levels, which may be due to the sequestration of CORT in the tissues by binding to the MR and GR receptors^35^ or due to local steroidogenesis and/or metabolism of CORT and 11DHC via 11HSDs. It was shown by others that hippocampus of rats exposed to chronic social defeat has higher level of CORT than cortex^24^ and that the density of hippocampal corticosteroid receptors is higher than in cortex ^36^. However, significant increase of brain CORT/PROG and CORT/11DOC ratios in stressed mice without any changes of corresponding plasma ratios (Figs. 3, Supplementary Fig. S2 online) and hippocampal ability to convert PROG to CORT^37^ give some evidence of local production of corticosterone. Additionally, high expression of 11HSD1 was reported in the hippocampus^38,39^. Thus, the rise of 11DHC due to 11HSD2 metabolism of peripheral CORT might further contribute to the increased brain CORT. The ratio CORT/11DHC, which reflects corticosterone regeneration and inactivation via 11HSDs, was significantly higher in the plasma and brain of stressed mice than in their colon, kidney, liver, and MLN. The low CORT/11DHC ratio and relatively high tissue 11DHC levels in the colon and kidney of stressed animals are consistent with the high activity of 11HSD2 even if both tissues express 11HSD1 and 11HSD2. Considering that 11HSD2 binds to its substrate with approximately 100 times more affinity than 11HSD1^14^, 11HSD2 may play a more dominant role in corticosteroid metabolism in tissues that coexpress both enzymes. The higher values of the CORT/11DHC ratio in brain areas and partially in the thymus than in other tissues associated with low tissue levels of 11DHC suggest different local regeneration or accumulation of corticosterone. Brain and lymphoid organs are tissues where 11HSD2 is absent or its activity is space-limited and very low compared to 11HSD1. These tissues show significant 11HSD1 activity, regenerating CORT from 11DHC^16,33,39^, and chronic social defeat upregulating the regeneration of CORT from 11DHC both in lymphoid organs^33^ and the hippocampus^40^. Although the contribution of blood steroids to the tissue levels cannot be excluded, Schliamser et al.^41^ showed that the blood content of the brain tissue varies between 0.26 and 0.7% and Little et al.^42^ demonstrated that the level of CORT in the hippocampus and cerebral cortex is not significantly influenced by saline perfusion prior to removal of the brain.

Stress induces not only hypercorticosteronemia but also upregulates plasma levels of PROG and 11DOC, the direct precursors of CORT via secretion from the adrenal glands^4,43,44^. We showed that stress increased the levels of PROG and 11DOC not only in plasma but also in other tissues. High levels of PROG and 11DOC in the brains of stressed mice, especially in the hippocampus, may indicate not only the accumulation of steroids from blood but also the de novo synthesis of neurosteroids in the brain^45,46^. Following acute swim stress, the levels of PROG and 11DOC in the hippocampus and other brain regions were increased in a manner similar to our results^5^. Similar to the brain, we observed increased PROG and 11DOC levels in the lymphoid organs of mice exposed to social defeat stress. However, one point to note is that, in contrast to brain tissue, the PROG/11DOC ratio in lymphoid organs was significantly increased and not decreased. It has been shown previously that thymus and some immune cells express upstream enzymes required for de novo steroidogenesis without the final glucocorticoid-synthetic enzyme Cyp11b1 and exhibit enzymatic activities associated with production of PREG, PROG and 11DOC^9,10,12,13,48^. In addition, the PROG/11DOC ratio was significantly higher in lymphoid organs than this ratio in plasma. Thus, the increased ratio in thymus and MLN may reflect a greater supply of circulating steroids and their sequestration in lymphoid tissues, including binding to appropriate receptors, or upregulation of local PROG synthesis either de novo from cholesterol or from circulating precursor. The elevated CORT/PROG and CORT/11DOC ratios in stressed animals likely reflect both differences in the sequestration of CORT, PROG and 11DOC synthetized de novo in the adrenal gland and transported by blood to the lymphoid organs but also the regeneration of CORT from 11DHC^16,47^.

Many studies have shown that stress is associated with an altered host microbiome; higher cortisol/corticosterone levels may reshape the microbial composition of the gut, and gut microbiota can alter the stress responses of the host^26,49,50^. Our results did not show any significant difference in the α-diversity of the fecal microbiome of stressed and unstressed mice, but we observed a significant difference in the β-diversity of the fecal microbiome. Similarly, the exposure of C57BL/6 and CD-1 mice to social stressor did not affect the α-diversity of the host gut microbiota but it changed the β-diversity significantly^49,50^. In contrast to mice, a human infant study did not prove any association between the cortisol stress response and β-diversity, even if α-diversity was weakly associated with the cortisol stress response^51^. The relative abundances of the major phyla and families were relatively similar between all groups, revealing the absence of a straightforward shift in the structure of the community, a finding observed previously in social defeated C57BL/6 mice^52^. However, LEfSe analysis identified several bacterial genera associated with stress, such as *Alistipes*, *Rikenella*, *Ruminiclostridium*, *Roseburia*, and *Helicobacter*. These findings are consistent with previous studies, which demonstrated enhanced colonization of the stomach by *Helicobacter pylori* in BALB/c mice exposed to psychological stress^53^, upregulated *Ruminoclostridium* in the fecal microbiome of heat-stressed rabbits^54^ and *Alistipes* in BALB/c mice exposed to grid floor chronic stress^55^ or repeated water immersion restraint stress^56^. Similarly, *Roseburia* and *Rikenella* were positively associated with stress in gut microbiome^25,57,58^. Additionally, positive correlation was observed between low corticosterone levels and low abundance of *Rikenella* in stressed rats^59^. However, in other studies, chronic restraint stress led to dramatic changes in colon microbiota at the phylum and genus levels, which was accompanied by decreased levels of *Rikenella*, *Roseburia*, and *Lachnospiraceae* and increased levels of *Prevotella*^50,60^. Whereas social stress reduced relative abundance of *Lactobacillus* in gut microbiota of CD-1 mice^49^, it increased relative abundance of *Lactobacillus* in BALB/c mice (Fig. 5C). This result correlates with the data of Xu et al.^61^, who showed increased levels of microbial metabolites including lactate in rats exposed to chronic stress. These observations indicate that stress effects microbiome diversity and that certain bacterial taxa can be considered as biomarkers related to stress exposure.

In conclusion, we explored local levels of corticosteroids in several tissue types in unstressed and chronically stressed male mice using an animal model of social defeat. The study demonstrated stress-dependent upregulation of CORT, 11DHC, PROG and 11DOC levels not only in plasma but also in brain and other tissues; however, the stress response was tissue-dependent and modulated the fecal microbial community. The findings indicate that the plasma level of corticosteroids may not always represent local levels in individual tissues and that exposure to corticosteroids during stress may be affected in a tissue-dependent manner. However, it leaves open the question of to what extent these differences are due to the extent of steroid enzyme activities and steroid accumulation in the tissue (degree of receptor binding, blood flow in tissue, tissue uptake).

## Supplementary Information

Supplementary Figures.

## Data Availability

The data produced during the current study can be made available from the corresponding author upon reasonable request or are deposited in the GenBank (sequencing data; accession no. PRJNA896122).
