# Supplementary material for: Profiling of adrenal corticosteroids in blood and local tissues of mice during chronic stress
Source: Sci Rep. 2023 May 4;13:7278. doi: 10.1038/s41598-023-34395-2 (PMC10160118; doi:10.1038/s41598-023-34395-2)
Supplement: Supplementary file 1 — Supplementary Figures. [file 41598_2023_34395_MOESM1_ESM.pdf]

Profiling of adrenal corticosteroids in blood and local tissues of mice during chronic stress

Karla Vagnerová
Michal Jágr
Chahrazed Mekadim
Peter Ergang
Hana Sechovcová
Martin Vodička
Kateřina Olša Fliegerová
Václav Dvořáček
Jakub Mrázek
Jiří Pácha

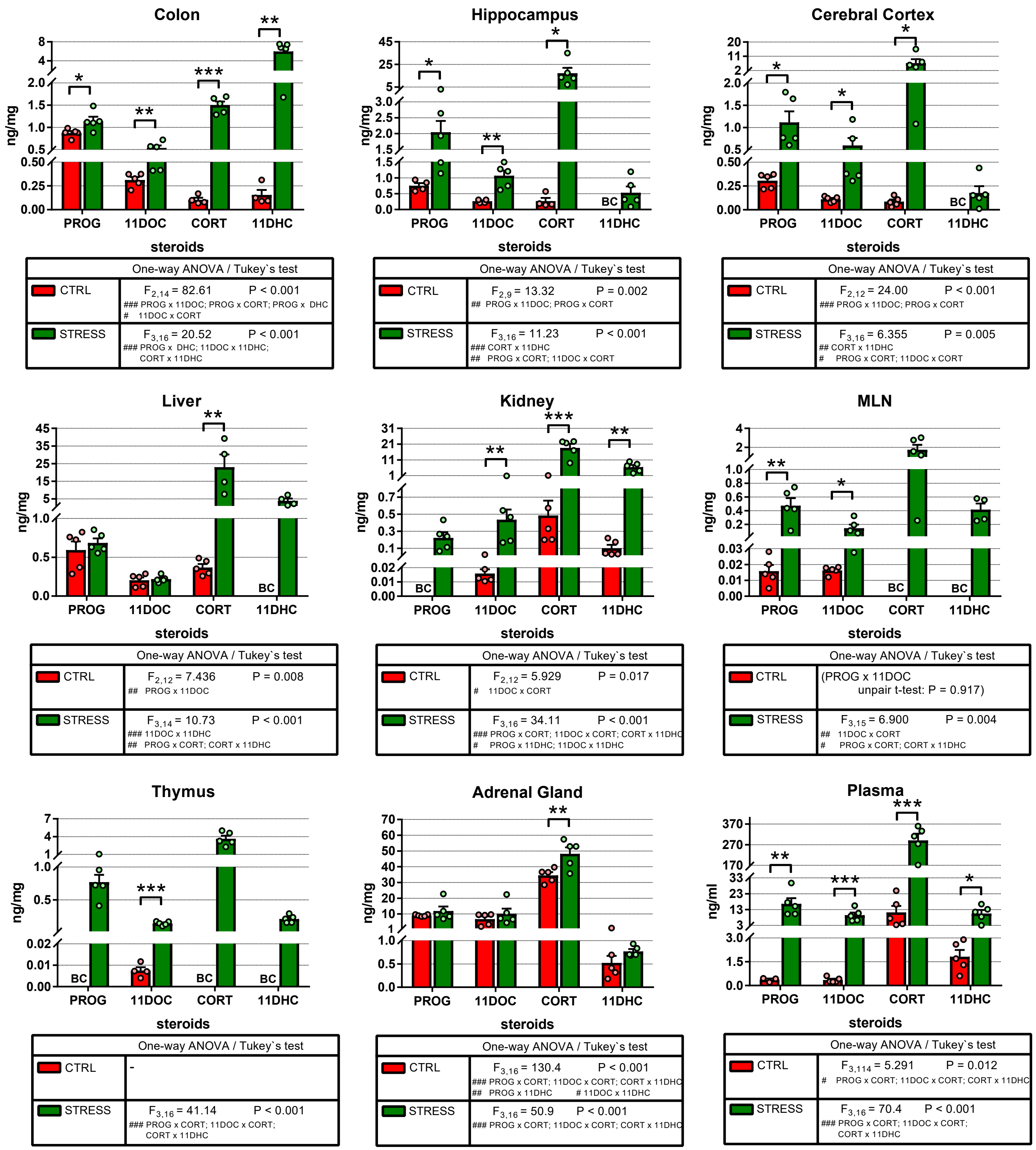

Supplementary Figure S1. Effect of social defeat on progesterone (PROG), 11-deoxycorticosterone (11DOC), corticosterone (CORT) and 11-dehydrocorticosterone (11DHC) level in the colon, brain, liver, kidney, lymphoid organs, adrenal gland and plasma. Red columns, control unstressed mice (CTRL); green columns, mice exposed to chronic social defeat (STRESS); MLN, mesenteric lymph node; BC, bellow the lowest value of the calibration curve. The amounts of steroids were converted to ng per ml of plasma or ng per mg of protein for other tissues. Data are shown as means ± SEM (n = 3-5). One-way ANOVA followed Tukey`s *post hoc* test were used to compare the steroid profiles in the respective tissues. Significantly different values between steroid levels: $^{###}P < 0.001$, $^{##}P < 0.01$, $^{#}P < 0.05$. Unpaired Student's t-test was used for analyzing the effect of repeated social defeat on the steroids levels in the individual tissues. Significantly different values between stressed and unstressed mice: ***P < 0.001, **P < 0.01, *P < 0.05.

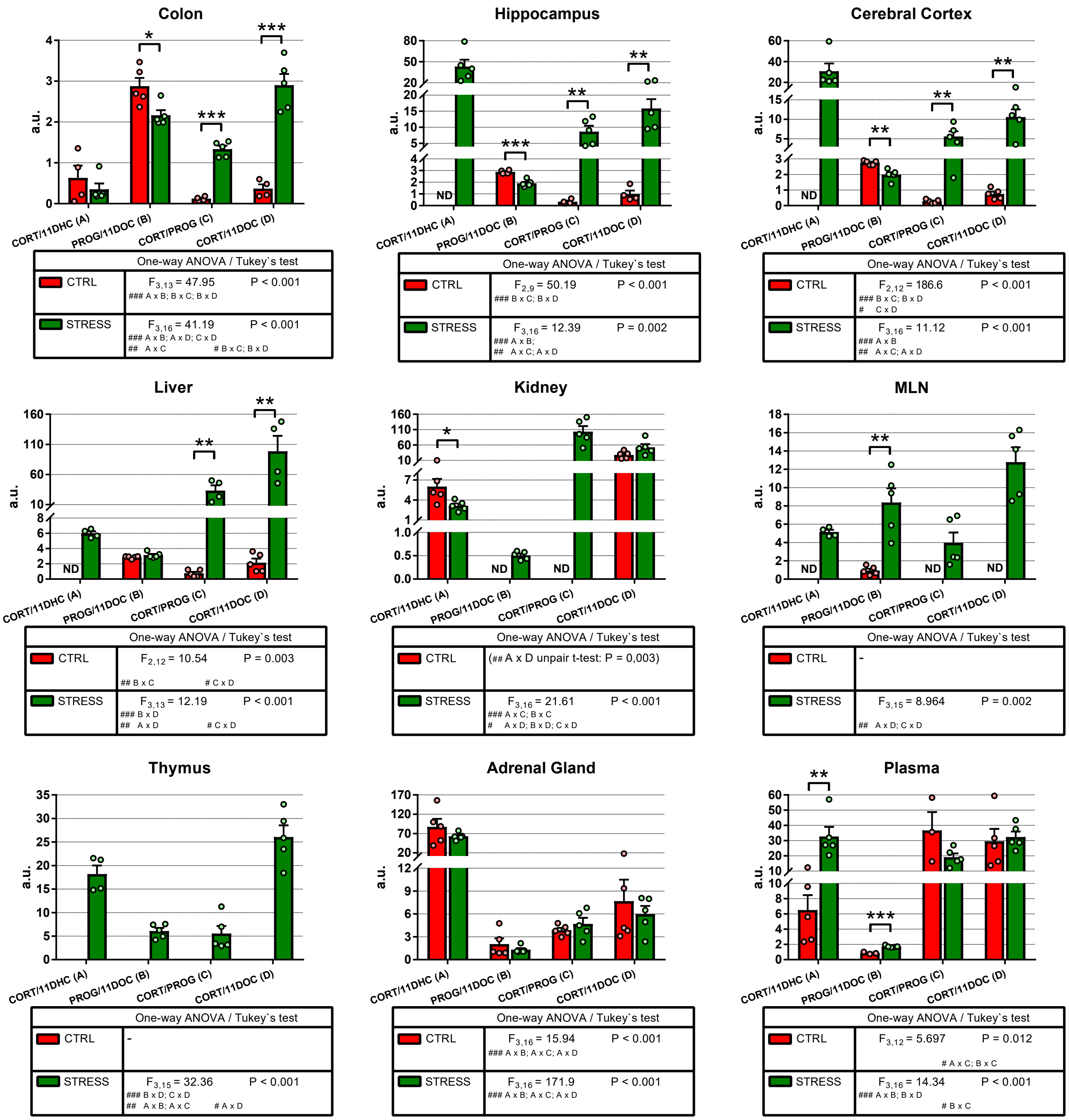

Supplementary Figure S2. Effect of social defeat on the corticosterone/11-dehydrocorticosterone (CORT/11DHC; A), progesterone/11-deoxycorticosterone (PROG/11DOC; B), corticosterone/ progesterone (CORT/PROG, C) and corticosterone/11-deoxycorticosterone (CORT/11DOC; D) ratio in the colon, brain, liver, kidney, lymphoid organs, adrenal gland and plasma. Red columns, controlled unstressed mice (CTRL); green columns, mice exposed to chronic social defeat (STRESS); MLN, mesenteric lymph node; ND, not determined. Data are shown as means ± SEM (n = 3-5). One-way ANOVA followed Tukey`s *post hoc* test were used to compare the steroids ratios in the respective tissues. Significantly different values between steroids ratios: $^{###}P < 0.001$, $^{##}P < 0.01$, $^{#}P < 0.05$. Unpaired Student's t-test was used for analyzing the effect of repeated social defeat on the steroids ratios in the individual tissues. Significantly different values between stressed and unstressed mice: ***P < 0.001, **P < 0.01, *P < 0.05.
